# Gut flora alterations among aquatic firefly *Aquatica leii* inhabiting various dissolved oxygen in fresh water

**DOI:** 10.1016/j.isci.2023.107809

**Published:** 2023-09-01

**Authors:** Zi-Shun Zhao, Lin-Yu Yang, Fu-Xin Li, Wei Cun, Xing-Yan Wang, Cheng-Quan Cao, Qi-Lin Zhang

**Affiliations:** 1 Faculty of Life Science and Technology, Kunming University of Science and Technology, Kunming 650500, China; 2 College of Life Sciences, Leshan Normal University, Leshan, Sichuan 614004, China

**Keywords:** Environment, Aquatic science, Microbiome

## Abstract

Knowledge about the impact of different dissolved oxygen (DO) on the composition and function of gut bacteria of aquatic insects is largely unknown. Herein, we constructed freshwater environments with different DOs (hypoxia: 2.50 ± 0.50, normoxia: 7.00 ± 0.50, and hyperoxia: 13.00 ± 0.50 mg/L) where aquatic firefly *Aquatica leii* larvae lived for three months. Their gut flora was analyzed using the combination of 16S rRNA amplicon sequencing and metagenomics. The results showed no difference in alpha diversity of the gut flora between *A. leii* inhabiting various DOs. However, the relative abundance of several bacterial lineages presented significant changes, such as *Pseudomonas*. In addition, bacterial genes with an altered relative abundance in response to various DOs were primarily related to metabolism. The alteration of these functions correlated with the DO change. This is the first to uncover structure of gut flora under various DOs in aquatic insect larvae.

## Highlights

• Dissolved oxygen (DO) impacted on the relative abundance of gut flora in *A. leii*
• The adjustment of metabolism-related function in gut flora plays a primary role
• Gut bacteria contributed to DO adaptation of the host
• We firstly characterized gut flora of aquatic insects inhabiting different DOs

## Graphical abstract

**Figure:**
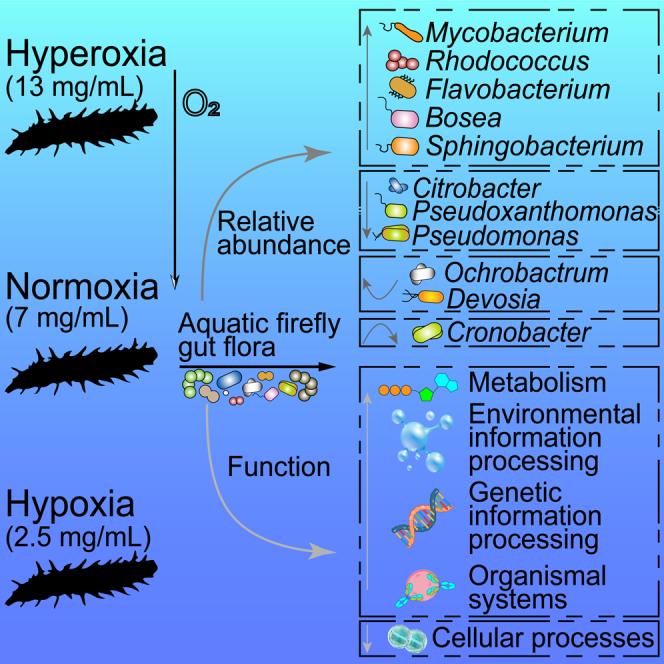

## Introduction

Compared with terrestrial lineages, aquatic animals face unique physiological challenges from the aquatic environment.^1^ In particular, the oxygen content in water (hereafter referred to as dissolved oxygen, DO) is much lower than that in air, being only ∼1/30 of that in air. DO levels in nature are unstable, the fluctuation is resulted by various reasons, such as degradation of organic pollutants, respiration of aquatic organism, and variation of water temperature.^2^ Fluctuation in DO levels was frequently monitored in some important ecological regions. For examples, DO concentrations range from 7.1 to 10.9 mg/L in coast of the Caspian Sea,^3^ and the concentration was reduced to lower than 2 mg/L in the northeast of Pacific Ocean and the north of Atlantic Ocean.^2^ As a major ecological factor, DO largely affects evolutionary and adaptive strategies of aquatic animals.^4^^,^^5^ A typical example is the gill as a key organ of respiration of aquatic animals. The development of the gill is affected by DO concentration. Previous studies found phenotypic plasticity of gill lamellae to different DO concentrations in goldfish.^6^ In particular, the inter lamellar cell mass of the gill lamellae were reduced or disappeared under hypoxia, and the surface area of gill lamellae increased approximately 7.5-fold, in comparison with normoxia, resulting in the improvement of the ability of gill to utilize fluctuating DO.

As the “second genome” of animals, gut flora has been reported to contribute to the adaptive evolution of animals to oxygen level. In particular, the abundance of gut flora is influenced by various oxygen concentrations in the environment where animals inhabit, and changes of the abundance of gut flora mediated changes in phenotypic characteristics, physiological metabolism, and behavior of the host. For examples, hypoxia pressure enhanced the metabolic level of *Rachycentron canadum*, with the increasing ratio of Bacteroidetes to Firmicutes in gut of the hosts.^7^ The abundance of *Bacillus* and *Lactobacillus* was significantly decreased in the gut of *Macrobrachium nipponense* due to hypoxic exposure, thereby increasing the susceptibility of the host to pathogenic bacteria.^8^ Hypoxia induced senescence of bone marrow mesenchymal stem cells via altered gut flora of patients with cyanotic congenital heart disease.^9^ Changes of the relative abundance of *Lactobacillus* were found in the gut of fruitfly under hypoxia, which affected mating and aggressive behavior of the host.^10^^,^^11^ These evidence indicated that oxygen levels have effects on structures of the gut flora behind the morphological changes of the host. Therefore, change in the structure of gut flora was a crucial adaptive feature to change in oxygen level in the host. However, exploration for effects of various DO concentrations on structures of the gut flora of the host behind aquatic adaptation is lacking, particularly for insects as the most abundant and diverse groups in the planet.

Several previous studies showed that effects of habitat and taxonomy of aquatic insects on gut flora. For examples, changes in composition of gut flora in mosquitoes larvae along with habitat alterations were found, and environment dominates over genetics in shaping the gut flora of mosquitoes.^12^ The lower diversity of gut bacteria Trichomycetes was found in aquatic insects (Culicidae, Simuliidae, Chironomidae, Ceratopogonidae (Insecta: Diptera), Ephemeroptera, and Plecoptera) inhabited at Misiones (a subtropical climate and rainforest vegetation, province of Argentina, the warmer temperatures of the water) compared to the colder streams of Tierra del Fuego, with forests and steppes as typical vegetation.^13^ In addition, significant differences were found in the relative abundances of gut anaerobes among insects that possessed different environments, diets (stenophagous, carnivorous, and herbivorous), developmental stages, and taxonomy.^14^ Therefore, habitat is a key in shaping the gut flora of insects, and investigation on effects of DO on gut flora of the host will expand the understanding of adaptation to aquatic habitat in insects.

The Lampyridae family (Insecta: Coleoptera), also known as fireflies, contains over 2,000 known species and 100 genera. Most firefly species (greater than 98%) are terrestrial and only nine species are aquatic.^15^^,^^16^ Only aquatic lineages exhibit adaptations to DO utilization in the larval morphology (e.g., branched tracheal gills and smooth and soft bodies) compared to terrestrial and semi-aquatic species.^16^^,^^17^ For examples, *Aquatica leii* larvae possess a smooth body surface, which contributed to reducing water resistance and aerobic respiration-based energy expenditure.^18^ In addition, several aquatic firefly lineages have up to eight pairs of gills, and the whole body is extremely extended when they rested on the bottom; meanwhile, the gills on the abdomen are fully expanded to increase the surface area of the gills,^18^ so as to increase the uptake of DO. Recently, transcriptomic and metabolomic analyses of fireflies revealed their molecular adaptations to ecological factors in fresh water, including DO.^19^^,^^20^ In particular, many genes involved in DO (e.g., those encoded NADH dehydrogenase, cytochrome *c* oxidase, and cytochrome P450) exhibited fast evolution and/or positive selection in aquatic firefly species. Metabolites involved in DO (e.g., gamma-glutamylleucine, cinchonidine, and pokeberrygenin) showed lineage-specific expression pattern in *A*. *leii*. The earlier morphological and genetic evidence indicated that aquatic fireflies are a suitable model for exploring DO adaptation in insects. However, to date, despite effects of DO on aquatic fireflies have been explored from the perspectives of molecular mechanism and morphology, studies on gut bacteria are still lacking. Even for all the insects, few studies have explored the altered gut flora in response to various DOs.

In this study, *A. leii* larvae chronically exposed to three different DOs (i.e., hypoxia, normoxia, and hyperoxia groups) were obtained. The gut flora of *A. leii* larvae inhabiting these three DOs was analyzed using the methods of 16S rRNA gene amplicon sequencing together with metagenomics to determine the effects of changes in DOs on gut flora, and provide candidates contributing to DO adaptation of the host.

## Results

### 16S rRNA amplicon sequencing

#### Data summary

**Figure 1:**
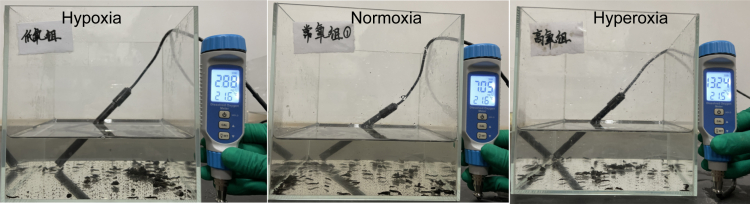
*A. leii* larvae inhabiting three groups of dissolved oxygen concentrations (DOs), i.e., hypoxia, normoxia, and hyperoxia groups

The summary of raw data were for 30 experimental samples treated by three different DOs (Figure 1) constructed in this study is provided in Table S1. In total, 717.2 M of raw data were obtained, with 1,584 tags assigned as operational taxonomic units (OTUs). Good coverage more than 99%, indicating that the majority of the bacterial phylotypes were identified in each sample. The number of OTUs per sample ranged from 32 to 99. The OTUs accumulation and rank curves (Figure S1) showed saturated and sufficient sequencing.

#### Composition of gut flora of A. leii under three DOs

**Figure 2:**
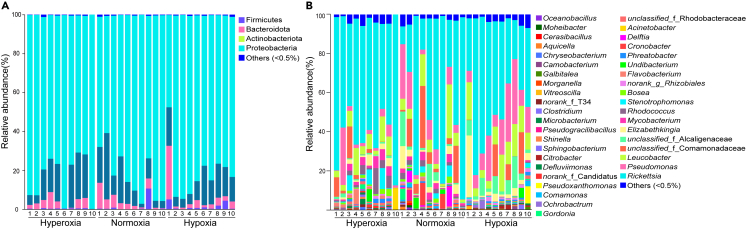
The relative abundance of the gut flora among the different groups (A and B) The relative abundance of the gut flora among the different groups at (A) phylum and (B) genus level. Stacked bars illustrate the abundance of clades and genera. Unclassified sequences are classified as “other”.

For the hypoxia group, five phyla, seven classes, 15 orders, 19 families, 20 genera, and 10 gut flora species were identified (Table S2). The dominant phylum was Proteobacteria (mean value in ten samples: 80.78%) (Figure 2A). Alphaproteobacteria (50.75%) were the most abundant bacteria at the class level (Figure S2A), followed by Gammaproteobacteria (30.03%). At the order level, Rickettsiales (45.6%) and Pseudomonadales (15.3%) were more abundant than the other lineages (Figure S2B). The predominant bacteria families were Rickettsiaceae (45.5%), followed by Pseudomonadaceae (15.2%) (Figure S2C). At the genus level (Figure 2B), *Rickettsia* (45.48%) and *Pseudomonas* (15.2%) dominated the gut flora, followed by *Leucobacter* (11.13%).

The normoxia group included five phyla, seven classes, 15 orders, 20 families, 19 genera, and 10 species. Proteobacteria were the most common bacterial phylum (81.92%) (Figure 2A). Alphaproteobacteria (54.81%) and Gammaproteobacteria (27.11%) were the dominant bacteria classes (Figure S2A). The major orders were Rickettsiales (51.54%) and Burkholderiales (15.12%) (Figure S2B). The predominant bacteria families were Rickettsiaceae (51.39%) and Pseudomonadaceae (8.25%) (Figure S2C). *Rickettsia* (51.39%), *Pseudomonas* (8.25%), and *Leucobacter* (7.64%) were the most abundant genera (Figure 2B).

For hyperoxia groups, five phyla, seven classes, 15 orders, 19 families, 19 genera, and nine species were identified. Proteobacteria (81.27%) were the most dominant phylum (Figure 2A). Alphaproteobacteria (67.58%) and Gammaproteobacteria (13.69%) were the top two bacterial classes (Figure S2A). Rickettsiales and Burkholderiales had higher relative abundances at the order level, with 62% and 5.19%, respectively (Figure S2B). The dominant bacterial families were Rickettsiaceae (61.32%), Microbacteriaceae (6.85%), and Pseudomonas (4.27%) (Figure S2C). Three genera, including *Rickettsia* (61.32%), *Leucobacter* (6.47%), and *Pseudomonas* (4.27%), presented the highest relative abundance (Figure 2B). At the species level, over 70% of the strains were not classified into one of the three DO groups (Figure S2D).

**Figure 3:**
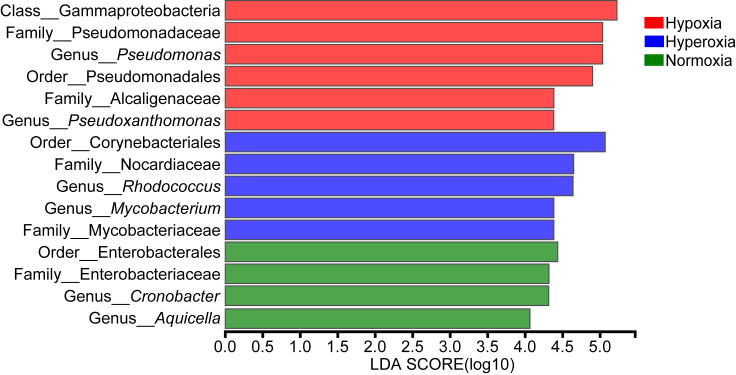
Histogram of LDA values.LDA scores for gut bacteria exceeding the threshold values of 4 indicate a significant difference between the groups. The *y* axis shows significant relative abundance difference compared to the other gut flora at each time point. Histogram length (LDA score) represents the effect size of gut bacteria

Linear discriminant analysis of effect size (LEfSe) showed that the taxa specific to the hypoxia group were *Pseudomonas*, while *Cronobacter* were specific to the normoxia group. The hyperoxia group was represented by the genera *Rhodococcus* and *Mycobacterium* (Figure 3; Table S3).

#### Discrepancies of A. leii gut flora under three DOs

**Figure 4:**
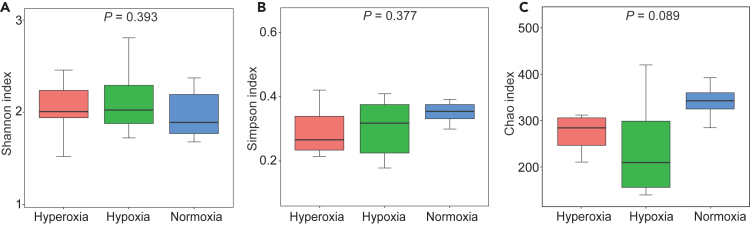
Alpha diversity analysis of the gut flora among the different groups based on the 16S rRNA gene sequences (A) Shannon index. (B) Simpson index. (C) Chao index.

**Figure 5:**
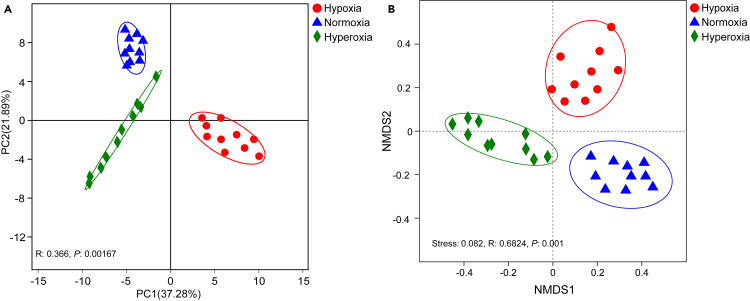
Cluster analysis of the samples based on the 16S rRNA gene sequences (A and B) Cluster analysis of the samples based on the 16S rRNA gene sequences in the principal component analysis (PCA) (A) and non-metric multidimensional scaling (NMDS) analysis (B).

The alpha diversity indices (i.e., Shannon, Simpson, and Chao) were not significantly different between any two of the three DO groups (Figure 4). The beta diversity analyses (principal component analysis (PCA) (Figure 5A) and non-metric multidimensional scaling [NMDS] (Figure 5B) analysis) presented obviously difference among different DO groups, consistently revealing cluster of samples from the same DO group but separate patterns among different DO groups, indicating different structure in the gut flora among DO groups.

**Figure 6:**
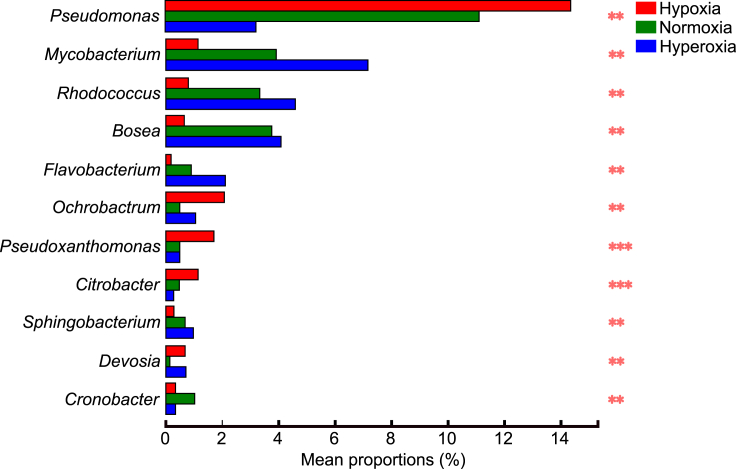
Multiple comparisons-based significance tests among the different groups based on the 16S rRNA gene sequences at genus level The significant differences (∗∗: < 0.01, ∗∗∗: < 0.001) were indicated by asterisks (Kruskal-Wallis test corrected by the Benjamini-Hochberg method).

The relative abundance of 11 bacterial genera was significantly different between the three DO groups (p < 0.05) (Figure 6). Table 1 lists their biological function. The relative abundance of three genera (i.e., *Pseudomonas*, *Pseudoxanthomonas*, and *Citrobacter*) decreased with an increase in DO levels. Conversely, the abundance of five genera (i.e., *Mycobacterium*, *Rhodococcus*, *Bosea*, *Flavobacterium*, and *Sphingobacterium*) showed an opposite trend. In addition, the relative abundance of *Cronobacter* in the normoxia group was significantly higher than in the other two DO groups, whereas that of two genera (i.e., *Ochrobactrum* and *Devosia*) was lower.

**Table 1:** Summary of the bacterial genera that responded to the change in the dissolved oxygen concentration

Family	Genus	Biological function
Pseudomonadaceae	*Pseudomonas*	Nutrition provision, antibiotic compound production, toxicant degradation, cellulose decomposition^21^^,^^22^
Mycobacteriaceae	*Mycobacterium*	N/A
Nocardiaceae	*Rhodococcus*	Degradation of polycyclic aromatic hydrocarbon, resorcinol reduction, parasite antagonism^23^^,^^24^
Boseaceae	*Bosea*	N/A
Flavobacteriaceae	*Flavobacterium*	Cellulose decomposition, lipid metabolism remodeling, improvement of lipid storage level and resistance to starvation^22^^,^^25^^,^^26^
Brucellaceae	*Ochrobactrum*	Pesticide degradation^27^
Lysobacteraceae	*Pseudoxanthomonas*	Neonicotinoid degradation, cellulose decomposition^28^^,^^29^
Enterobacteriaceae	*Citrobacter*	Promotion of gut histological changes, immunity regulation, cellulose decomposition^22^^,^^30^
Sphingobacteriaceae	*Sphingobacterium*	Plastic degradation^31^
Devosiaceae	*Devosia*	Cellulose decomposition^22^^,^^32^
Enterobacteriaceae	*Cronobacter*	N/A

### Metagenomic sequencing

#### Overview of sequencing data

Clean data of 40.71 Gb, 38.54 Gb, and 37.88 Gb were obtained for the hypoxia, normoxia, and hyperoxia groups, respectively (Table S4). Moreover, 11,789,946 contigs with an average N50 value of 733.56 bp for each sample were obtained after assembly, revealing a robust assembly for metagenomes.

#### Analysis of composition and relative abundance of A. leii gut flora under three DOs

**Figure 7:**
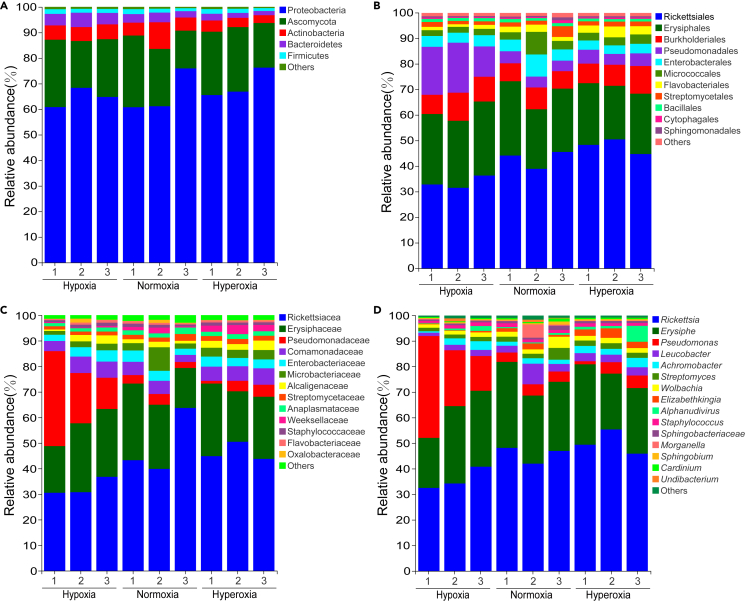
The relative abundance of the gut flora among the samples (A–D) At (A) phylum, (B) order, (C) family and (D) genus levels based on metagenomic sequencing.

Proteobacteria was the dominant phylum in *A. leii* gut under all three DO groups, accounting for 64.62% (average values of three replicates), 65.93%, and 69.52% of the hypoxia, normoxia, and hyperoxia group samples, respectively (Figure 7A). The Rickettsiales order was the most abundant, accounting for 33.64%, 42.96%, and 47.92% of the hypoxia, normoxia, and hyperoxia groups, respectively (Figure 7B). Rickettsiaceae family was the most abundant, which accounted for 46.37%, 32.68%, and 48.94% in the hypoxia, normoxia, and hyperoxia groups, respectively (Figure 7C). At the genus level (Figure 7D), *Rickettsia* (50.31%) (p value = 0.025), *Achromobacter* (2.94%) (p value = 0.038), and *Elizabethkingia* (2.91%) (p value = 0.036) showed greater relative abundances in the hyperoxia group than in the other two groups. Compared with the remaining two groups, the relative abundances of *Leucobacter* (4.56%) (p value = 0.024), and *Wolbachia* (2.67%) (p value = 0.013), were higher in the normoxia groups. The relative abundance of *Pseudomonas* (25.09%) in the hypoxia group was higher than in the other two groups. Pearson correlation coefficient of the relative abundance at the genus level between 16S and metagenomic analysis was 0.96, p < 0.001.

#### Potential function of gut flora

Based on the database of Clusters of Orthologous Groups (COG), 14,490 genes were annotated from all the samples, and classified into 19 functional categories. These categories included carbohydrate transport and metabolism (9.7%), translation, ribosomal structure and biogenesis (8.2%), and amino acid transport and metabolism (6.3%). For the Kyoto Encyclopedia of Genes and Genomes (KEGG), 9,080 genes were annotated, and divided into six categories, 23.7% of which were assigned to metabolism, followed by 13.6% belonging to environmental information processing. Based on the non-Redundant (NR) database, 1,677 genes were annotated, and most genes were related to fold hydrolase, amino acid permease, and carboxylesterase. In total, 244 genes were annotated in the carbohydrate-active enzymes (CAZy) database, involving glycoside hydrolase (101), glycosyl transferase (66), carbohydrate-esterase (11), carbohydrate-binding plate (16), and polysaccharide lyase (19).

**Figure 8:**
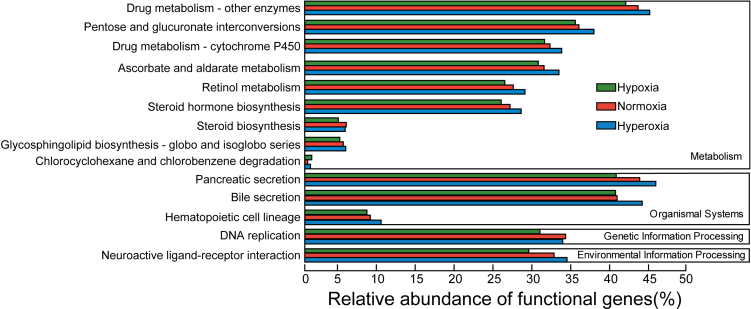
Differential functions among the hypoxia, normoxia, and hyperoxia groups based on the Kyoto Encyclopedia of Genes and Genomes (KEGG) annotation of genes

A significant difference (p < 0.05) in the function of the gut flora was detected among *A. leii* inhabiting different DOs, including metabolism, genetic information processing, organismal systems, and environmental information processing (Figure 8; Table S5). In particular, genes related to the degradation of chlorocyclohexane and chlorobenzene showed higher (p value = 0.028) relative abundance in the hypoxia group than in the other two groups; genes related to steroid biosynthesis showed higher (p value = 0.032) relative abundance in the normoxia group. In the hyperoxia group, genes associated with drug metabolism-other enzymes, pentose and glucuronate interconversions (p value = 0.048), drug metabolism-cytochrome P450 (p value = 0.034), ascorbate, and aldarate metabolism (p value = 0.013), retinol metabolism (p value = 0.021), steroid hormone biosynthesis (p value = 0.038), glycosphingolipid biosynthesis-globo, and isoglobo series (p value = 0.015) presented more relative abundance.

#### Correlation of the change in the relative abundance of the functional genes in the gut flora with DO alteration

**Figure 9:**
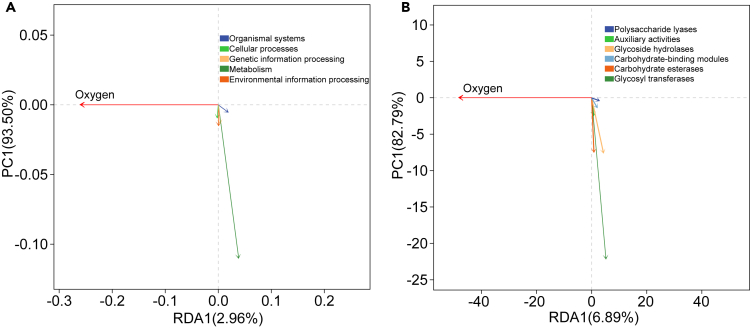
The correlation between the function and DO variation (A and B) Analysis of the correlation between the function (A): KEGG annotation and (B): CAZy annotation of the gut flora and dissolved oxygen using RDA. The environmental factors/function are represented by arrows. The different colors of the arrows represent the different functional genes. The length of the arrow connecting the lines represents the degree of correlation between the environmental factors and the functional genes; the longer the line, the greater the correlation, and vice versa, the smaller the correlation. The angle between the arrows represents the positive and negative correlation between the environmental factors and the functional genes (acute angle: positive correlation; obtuse angle: negative correlation).

Redundancy analysis (RDA) revealed that the KEGG function of genes (Figure 9A) was assigned to five categories significantly related to DO variation. Notably, the genes involved in metabolism exhibited a strong negative correlation with DO variation. The genes involved in genetic information processing, environmental information processing, and organic systems showed a negative correlation. However, there was a positive correlation between DO variation and the genes involved in cellular processes. According to the results of RDA implemented in the CAZy database (Figure 9B), the genes involved in glycosyltransferase showed a strong negative correlation with DO variation. The genes involved in carbohydrate esterases and glycoside hydrolases presented a moderately negative correlation. In contrast, the genes involved in auxiliary activities, carbohydrate-binding modules, and polysaccharide lyase showed a negative correlation.

## Discussion

In this study, the composition of the gut flora of *A. leii* larvae inhabiting three DOs was analyzed using a metagenomics approach combined with 16S rRNA gene sequencing. A high and significant correlation of the results obtained by 16S analysis and metagenomic methods indicated the robustness of analyses implemented in this study, with only a slight inconsistency in the relative abundance of the gut flora (e.g., *Erysiphe*, *Wolbachia*, and *Sphingobacterium*) detected. This pattern has been frequently observed in earlier studies, which is attributed to the fact that 16S rRNA gene amplicon sequencing captures a small sequence segment and is influenced by PCR primer.^33^^,^^34^ Although alpha diversity in the gut flora of *A. leii* larvae was found to be unchanged with the change in the DOs in this study, the relative abundance of different gut flora presented a change. This is the first study to show effects of DO on the relative abundance of the gut flora in aquatic animals rather than diversity.

In particular, this study found a change in the relative abundance of Proteobacteria and Actinobacteria, as two dominant phyla in *A. leii* gut, with DOs, as similarly reported in the gut of *Litopenaeus vannamei* and *Macrobrachium rosenbergii*.^35^^,^^36^ In the insect, Proteobacteria and Actinobacteria metabolize secondary metabolites from food to help the host digestion, and promote the host growth and development, and resist the infection.^37^^,^^38^^,^^39^^,^^40^ In addition, the relative abundance of Bacteroidetes and Firmicutes phyla also changed with the DOs in the current study. The ratio of the former to the latter was obviously higher in the hyperoxia group (11.00) than in the hypoxia group (4.83) and the normoxia group (3.41). Bacteroidetes can degrade carbohydrates and proteins to facilitate their absorption by the host.^41^^,^^42^ A higher ratio of Bacteroidetes to Firmicutes in the gut flora has a greater absorption of food energy by the host.^43^ Therefore, Bacteroidetes possibly promoted the adaptation of *A. leii* larvae to DO by adjusting the nutrition absorption of food by the host. Moreover, *A. leii* larvae need high energy metabolism under a hyperoxia environment, which might be beneficial with a higher ratio of Bacteroidetes to Firmicutes. Overall, the aforementioned phyla may help the host to effectively adjust absorption of nutrition substances and maintain balance between energy production and metabolism of the host under various DOs.

At the genus level, the current study showed that the relative abundance of *Pseudomonas* and *Mycobacterium* significantly changed with the DOs. These two bacterial genera are antagonistic.^44^ As a typical aerobic pathogen, *Mycobacterium* has been widely found in the soil, mammals, and bugs.^45^ A decreasing trend of the relative abundance of *Mycobacterium* with the decreasing DOs may be explained by its aerobic characteristics and/or antagonism caused by other bacteria (e.g., *Pseudomonas*). *Pseudomonas* engaged with varied interactions with insects, being either a pathogen or beneficial endosymbiont and using insects as vectors.^46^ Despite *Pseudomonas* being also aerobic lineage, its relative abundance increased with the decreasing DOs. It can provide essential nutrients (i.e., amino acids, cofactors, and vitamins) to the host under nonpathogenic conditions.^46^ Furthermore, this genus can produce antibiotic compounds, degrade toxic substances, convert insoluble substances, and decompose organic substances to protect insects from adverse circumstances (e.g., microbial infection).^21^ This finding indicated that *Pseudomonas* present in the gut of *A. leii* larval probably protect the host from hypoxia injury or pathogenic bacteria infection (e.g., *Mycobacterium*) caused by DO change.

In addition, change in DO concentration induced the increasing content of ammonia of blood via affecting nitrogen metabolism of aquatic animals.^47^^,^^48^ *Rhodococcus* and *Sphingobacterium* presented an alteration in the relative abundance of *A. leii* larval gut, which is known to degrade harmful substances (e.g., ammonia nitrogen), resist parasites, and enhance immunity in host gut.^23^^,^^31^^,^^49^ This implies that *Rhodococcus* and *Sphingobacterium* may help adjust the capacity of detoxification and immunity of larval *A. leii* exposed to different DO environments. Four bacterial lineages (i.e., *Flavobacterium*, *Pseudoxanthomonas*, *Citrobacter*, and *Devosia*) which function as cellulose degradation, lipid metabolism adjustment, and resistance improvement for the host to starvation^22^^,^^25^^,^^28^ were found to be altered in the relative abundance in this study. This may help the host balance the nutrient uptake and energy consumption level in *A. leii* larvae suffering from altered DO. The current study also detected a change in the relative abundance of *Ochrobactrum* and *Pseudoxanthomonas* in *A. leii* larval gut among the DOs. Previous studies reported the pesticide and nicotine degradation function of *Ochrobactrum* and *Pseudoxanthomonas*,^27^^,^^29^ indicating that these two lineages protect larval *A. leii* from oxygen poisons (the compromised function of mitochondria and membrane damage caused by oxidative stress) under the DO change.

The relative abundance of metabolism-related genes in the gut flora of *A. leii* larvae presented a change with different DOs. In particular, genes involved in pentose and glucuronide interconversions were reported to contribute to high-altitude adaptation of yaks by adjusting sugar and carbohydrate metabolism.^50^ Therefore, a change in the relative abundance of genes related to pentose and glucuronide interconversions may improve carbohydrate metabolism in the gut flora of *A. leii* larvae under various DOs. Expression change of cytochrome P450 genes widely reported to be associated with detoxification and antioxidation in insects^20^ were susceptible to altered DO.^51^^,^^52^ The current study found changes in the relative abundance of genes involved in drug metabolism-cytochrome P450, suggesting that improving gut flora’s detoxification and antioxidation capacity is essential for the host resistance to oxidative stress from altered DOs. Steroid hormones are involved in several physiological processes such as energy metabolism, maintenance of cellular sodium-potassium homeostasis, and insect molting.^53^^,^^54^^,^^55^ In this study, changes in the relative abundance of genes related to steroid hormone biosynthesis indicated that steroid hormones-mediated cellular homeostasis, development, and growth of the host might benefit from the gut flora. As a typical antioxidant, only low-content ascorbic acid can eliminate reactive oxygen species (ROS),^56^ contributing to the resistance of *Dicentrarchus labrax* to hypoxic injury.^57^ This evidence indicated that gut flora promoted ascorbic acid-based ROS elimination when the host suffered from unusual DOs. The retinol (vitamin A) is oxidized into retinoic acid, which plays important roles in cell growth, differentiation, and organogenesis.^58^ Excessive retinol caused tissue hypoxia and pathological endosteum mineralization in rats,^59^^,^^60^ but retinol metabolism was an essential regulator for retinol levels in the hypoxia response of silver sillago.^61^ Thus, gut flora involved in retinol metabolism may contribute to normal cell development and avoidance of paradoxical oxygen damage in *A. leii* larvae by maintaining normal retinol levels under various DOs. In addition, changes in the relative abundance of genes related to DNA replication and neuroactive ligand-receptor interaction were detected in the current study. Enhancement or diminishment of this genetic and environmental information processing regulated by the gut flora promotes well the host to cope with extreme DO, as genes related to DNA replication contributed to high-altitude hypoxia adaptation of grassland caterpillars, and neuroactive function promoted hypoxia tolerance in brain tissue of silver carp.^62^^,^^63^ Pathways involved in aquatic insect larvae, such as pancreatic secretion, play a crucial role in vertebrates. However, homologous genes in these pathways were found in the present study to contribute to the survival of *A. leii* larvae under altered DOs. Indeed, due to lacking higher organs/tissues (e.g., pancreas, liver, and bone marrow) in insects compared to vertebrates, these gut flora genes displayed different function targets between vertebrates and insects. Nevertheless, it is also possible to target homologous tissues (e.g., fat body and hemolymph) in insects.

Moreover, RDA indicated that enhancement of the metabolism, genetic function, environment adaptation ability, and immunity of the gut flora, with the decreasing cellular processes, likely help *A. leii* larvae to cope with the decreasing DO. More or less, these gut flora functions have been reported in lizards and *Cyprinodon variegatus* under hypoxia^64^^,^^65^ and in humans.^66^ In particular, a strongly negative correlation between the abundance change in genes related to metabolism with DO variation further demonstrated the core of metabolism function when the host suffered from extreme DO. In addition, RDA also uncovered the great significance of gut flora-mediated carbohydrate metabolism processed by six enzyme categories identified here for the survival of host chronically exposed to extreme DO, particularly glycosyltransferase, because the carbohydrate utilization efficiency of *A. leii* larvae could be improved by the enhanced functions of the gut bacteria.^33^

In summary, this study examined fresh water with various DOs to treat aquatic firefly and obtained large-scale sequencing data of the gut flora. *A. leii* larvae survived for long periods in fresh water with different DOs, significantly altering the relative abundance of the key gut microbiota. These bacterial lineages likely help the host to cope with altered DOs by adjusting the biological function primarily involved in metabolism, organismal systems, and genetic and environmental information processing.

### Limitations of the study

Some targeted genes and gut bacteria (e.g., *Cronobacter* and *Bosea*) have no functional information and/or cannot be linked to response of *A. leii* larvae to the altered DO. Nonetheless, these targets indeed have presented key roles that helped the host better to cope with various DOs. Further investigations using functional genomics and *in vivo* studies in *A. leii* larvae are required to confirm the functional genes and bacterial lineages identified in this study. In addition, the scope of the current study’s results among aquatic insects and even animals remains largely unknown. Therefore, the targeted genes and gut bacteria must be confirmed in more aquatic lineages, which is valuable work for understanding the aquatic adaptation of animals from the gut flora perspective.

## STAR★Methods

### Key resources table

**Table:** 

REAGENT or RESOURCE	SOURCE	IDENTIFIER
**Biological samples**		

*Aquatica leii* larvae	Kunming University of Science and Technology	*Aquatica leii*

**Chemicals, peptides, and recombinant proteins**		

Oxygen	Kunming NingQun	N/A
Nitrogen	Kunming NingQun	N/A
0.2 μm PTFE film	Green Mall	N/A
75% Ethanol	ChemicalBook	CAS: 64-17-5
Agarose	TsingKe	Cat.No.TSJ001
Fast Pfu DNA polymerase kit (with dNTP) 250U	HuanKai Biology	HKE031-01B

**Critical commercial assays**		

DNA extraction Kit	Tiangen	Cat. #DP302-02
AxyPrep DNA Gel Extraction Kit	AxyPrep	Cat. #GX-250
TruSeq® DNA PCR-Free Sample Preparation Kit	Illumina	FC-121-3002
NexteraXT DNA Library Preparation Kit	Illumina	FC-131-1096
TIANquick Midi purification Kit	Tiangen	Cat. #DP204

**Deposited data**		

16S rRNA amplicons sequencing data	NCBI SRA database	PRJNA869514
metagenomic sequencing data	CNCB GAS database	PRJCA012698

**Oligonucleotides**		

16S-338F	Majorbio	5′-ACTCCTACGGGAGGCAGCAG-3′
16S-806R	Majorbio	5′-GGACTACHVGGGTWTCTAAT-3′

**Software and algorithms**		

FLASH	https://ccb.jhu.edu/software/FLASH	V1.2.11
USEARCH	http://www.drive5.com/usearch	V11
UCHIME	http://drive5.com/uchime/uchime_download.html	V4.2.40
RDP classifier	https://sourceforge.net/projects/rdp-classifier/	V2.2
R Statistical software	https://www.r-project.org/	V3.1.1
Mothur	http://mothur.org/	
QIIME2	https://qiime2.org/	N/A
FastQC	https://www.bioinformatics.babraham.ac.uk/projects/fastqc/	V0.11.9
IDBA-UD	https://github.com/loneknightpy/idba	
Bowtie 2	https://bowtie-bio.sourceforge.net/bowtie2/index.shtml	V2.4.5
MetaGeneMark	http://topaz.gatech.edu/GeneMark/license_download.cgi	V3.25
CD-HIT	https://github.com/weizhongli/cdhit/releases	V4.6.1
SOAPaligner	https://help.rc.ufl.edu/doc/SOAPaligner	V2.21
STAMP	https://beikolab.cs.dal.ca/software/STAMP	V2.1.3

**Other**		

Spectrophotometer	Thermo Scientific	ND-2000
PCR thermocycler	ABI GeneAmp	9700
Fluorometer	Quantus	N/A
Focused ultra-sonicator	Covaris	ME220
Bioanalyzer	Agilent	2100
High throughput sequencer	Illumina Hiseq	X Ten

### Resource availability

#### Lead contact

Further information and requests for resources and reagents should be directed to and will be fulfilled by the Lead Contact, Qi-Lin Zhang (zhangql@kust.edu.cn). The dataset is not declared to be publicly accessible.

#### Materials availability

This study did not generate new unique reagents.

### Experimental model and study participant details

The developmental stages of firefly lineages include eggs, larvae, pupae, and adults. According to larvae habitat preferences, firefly species can be divided into terrestrial, aquatic (i.e., *Luciola* and *Aquatica*), and semi-aquatic lineages.^15^^,^^16^ In this study, aquatic *A. leii* larvae were originally obtained from the Culture Center of Fireflies, Ganzhou, Jiangxi Province, China, kept at the Faculty of Life Science and Technology, Kunming University of Science and Technology. Larval *A. leii* possesses six instars, with approximately 300 days at the whole larval stage, and each of the fourth and the fifth instar span for nearly 50 days.^18^^,^^67^ In total, 1,200 healthy fourth instar larvae were randomly selected and transferred to 15 L glass jars for ten days to adapt to the experimental environments. During this period, the water body was maintained at an approximate 7 mg/L concentration of dissolved oxygen at 21 ± 1°C, and range of pH values was 6.5–7.0, replacing one-third of the water every two days, and the exuvium generated after molting was removed. Feeding for *A. leii* larvae was maintained to avoid abnormal physiology and death. Muscle tissue (25 g per group) of the freshwater planorbid snail (*Gyraulus convexiusculus*) was fed to *A. leii* larvae every 48 h. The gender of firefly larvae has not been distinguished in prior research, and gender does not influence the outcomes of this investigation.

### Method details

#### Treatment of different dissolved oxygen concentration

The experimental individuals were randomly divided into three 5 L glass jars containing three different DO as the experimental groups. Each tank/treatment contained approximately 400 individuals, and each larvae from the same tank was considered as a replicate. The glass jars were covered with an aseptic film. DO levels were maintained by bubbling mixed gas in different ratio of pure nitrogen and oxygen. The hypoxia, normoxia, and hyperoxia groups were assigned to water bodies with DO values of 2.50 ± 0.50 mg/L, 7.00 ± 0.50 mg/L, and 13.00 ± 0.50 mg/L, respectively. The levels of DO were monitored using a dissolved oxygen meter (REX, China), recorded every 2 h for three months (Figure S3). The *A*. *leii* larvae were raised in a tank with pure water, kept clean by periodically removing contaminants using a mesh pocket with handles. Therefore, the effects of contamination or waterborne on experimental individuals can be omitted during the whole experiment.

#### Sampling and DNA extraction

In order to obtain resident gut flora rather than transient that from foods, the experimental subjects were starved for five days prior to use. Ten individuals of the sixth instar larvae were randomly selected from each DO group for further DNA extraction. After washing with 75% ethanol for 70 s and three rinses with ddH_2_O, the midguts were collected separately for each DO group. Integrity and purity of the genomic DNA extracted using a DNA extraction kit (Tiangen, China) were assessed using 1.2% agarose gel electrophoresis and a NanoDrop ND-2000 spectrophotometer (Thermo Scientific, USA).

#### Sequencing and analysis of the V3-V4 region of the 16S rRNA gene

The hypervariable region V3-V4 of the bacterial 16S rRNA gene was amplified with primer pairs 338F (5′-ACTCCTACGGGAGGCAGCAG-3') and 806R (5′-GGACTACHVGGGTWTCTAAT-3') using an ABI GeneAmp 9700 PCR thermocycler (ABI, USA). The PCR reaction mixture included 4 μL 5 × Fast Pfu buffer, 2 μL 2.5 mM dNTPs, 0.8 μL each primer (5 μM), 0.4 μL Fast Pfu polymerase, 10 ng of template DNA, and ddH2O to a final volume of 20 μL. PCR amplification cycling conditions were as follows: initial denaturation at 95°C for 3 min, followed by 30 cycles of denaturation at 95°C for 30 s, annealing at 55°C for 30 s, extension at 72°C for 45 s, and final extension at 72°C for 10 min. All samples were independently amplified in triplicate. The AxyPrep DNA Gel Extraction Kit (Axygen Biosciences, USA) was used to purify the PCR products, and the Quantus Fluorometer (Promega, USA) was used to quantify them. Sequencing libraries were generated using a TruSeq DNA PCR-Free Sample Preparation Kit (Illumina, USA) under quality control. Finally, the purified amplicons were pooled in equimolar. Their paired-end sequencing (2 × 300 bp) was conducted on an Illumina MiSeq platform under the standards at Majorbio Bio-Pharm Technology Co., Ltd. (Shanghai, China).

Raw FASTQ files were filtered by discarding reads with mononucleotide repeat length ≥10 bp, without overlap (overlap = 15 bp), and containing N-base. First, FLASH software (v1.2.11)^68^ was employed to assemble clean reads according to their overlap sequence. Then, using a minimum length of 15 bp and a mismatch rate of 0.1, low-quality assembled tags were removed. Following that, tags were aggregated into OTUs using USEARCH software (97% sequence identity).^69^ UCHIME (v4.2.40) was used to filter the chimeras generated by PCR amplification from OTUs.^70^ Finally, with confidence scores of ≥ 0.6,^33^ species classification annotation of representative OTU sequences was obtained using the RDP classifier (v2.2) according to the Silva 138.1 SSU reference databases (released data: August 2020).^71^

An OTU-based Venn diagram, as well as rank and species accumulation curves, were plotted using R. For alpha diversity analysis, the number of OTUs, Shannon, Simpson, Chao, ACE, and cover indices were calculated using mothur^72^ and plotted using R. Beta diversity was assessed by cluster (PCA and NMDS) analysis in QIIME2,^73^ and statistical test was performed using the ANOSIM analysis with Euclidean algorithm. Finally, LEfSe (LDA >4) analysis was employed to identify specific bacterial lineages among DOs.

#### Sequencing and analysis of metagenomics

Larval *A. leii* individuals used in metagenomics were different from that of 16S analysis, and three homogenates were used per DO treatment. A mixture of midgut DNA from twenty individuals was used for metagenomics analysis of *A. leii* larvae gut flora from each of the three DOs. The construction and sequencing of the metagenomic library were conducted according to the standard protocols by Majorbio (Shanghai, China). In short, validated DNA samples were fragmented by sonication using a Covaris ME220-focused ultra-sonicator (Covaris, USA). The DNA fragments were purified using a TIANquick Midi purification kit (Tiangen) after terminal-end repair. An "A" base was linked to the 3' terminal of the purified fragments, and a sequencing adaptor was linked to the 3' and 5′ terminals. A DNA purification kit was used to select DNA fragments of suitable sizes following quality control. To isolate and purify the PCR amplicons of the target DNA segments, 2% agarose gel electrophoresis and a DNA purification kit were used. The quality of the sequencing libraries was determined using an Agilent 2100 Bioanalyzer (Agilent Technologies, USA). Finally, nine sequencing libraries (three DOs × three biological replicates) were sequenced on an Illumina HiSeq X Ten platform.

Data pre-processing was conducted using FastQC software (v 0.11.9) (https://www.bioinformatics.babraham.ac.uk/projects/fastqc/), which was used during quality control to check raw sequence data. Low-quality raw reads (with low quality [Q ≤ 5] value >50%, N bases >10%, and adaptor) were discarded. Reads aligned to the insect genome using SOAP2 software^74^ were further removed. Then, IDBA-UD software^75^ was used to simultaneously assemble quality clean reads into contigs under multiple k-mer parameters for each sample. The assembly quality of the contigs was determined using SOAP2 software. Contigs beyond 300 bp were retained for open reading frame prediction in MetaGeneMark (V3.25).^76^ CD-HIT v 4.6.1 software (90% sequence identity) was used to remove redundancy in the contigs and generate final gene sets using.^77^ Using SOAP aligner v 2.21,^78^ the acquired genes were aligned to non-redundant gene catalogs to determine gene abundance with 95% identity, and further annotation was carried out using default parameters. Moreover, in order to obtain taxonomic information on gut flora, the above obtained 16S gene set was searched in the non-redundant protein sequence (NR) database of NCBI with DIAMOND software^79^ (v0.28.22.84) using default parameters for BLASTx comparison and an e-value of 1e-5, and taxonomic information was extracted according to the NR annotation information.

To investigate the potential function of gut flora in *A. leii* at different DO, the above-predicted gene set was searched in the following databases using BLAST software with default parameters, including COG (https://www.ncbi.nlm.nih.gov/COG/), Swiss-Prot (https://www.uniprot.org/), KEGG (https://www.kegg.jp/), and CAZy (http://www.cazy.org/). Based on KEGG annotation, the difference in the relative abundance of the genes in each functional term in the gut flora among *A. leii* inhabiting the three DO was generated using STAMP v2.1.3 software.^80^ In addition, genes were searched in the CAZy database to identify those involved in the digestive enzymes. Moreover, RDA analysis between the environmental factors and gene function (i.e., digestive enzymes and KEGG pathway) was performed, with significance levels calculated by permutest analysis, to explore further the effect of DO variation on the gut flora function.

### Quantification and statistical analysis

The differences of alpha diversity indices were analyzed using Kruskal-Wallis test method. Significance test was conducted using Kruskal-Wallis test for the other multiple comparison in this study; moreover, the Benjamini-Hochberg adjustment was employed as the correction method of a false discovery rate (*FDR*). Statistical analysis was performed in IBM SPSS Statistics 22. The significance levels in the difference in the relative abundance of the genes in each KEGG term among three DO groups were assessed by one-way ANOVA plus Bonferroni post-tests, corrected by the Benjamini-Hochberg method (*FDR*, the threshold is 0.05).

## Data Availability

• The 16S rRNA amplicons sequencing data generated in this study have been deposited in the NCBI SRA database (accession number: PRJNA869514). The metagenomic sequencing data have been deposited in the CNCB GSA database (accession number: PRJCA012698). These data are publicly available. • All relevant data supporting the findings of this study are available from the lead contact upon request. • The published article and supplemental information include all data generated and analyzed during this study. This paper does not report original code.
