# Supplementary material for: Gut flora alterations among aquatic firefly Aquatica leii inhabiting various dissolved oxygen in fresh water
Source: iScience. 2023 Sep 1;26(10):107809. doi: 10.1016/j.isci.2023.107809 (PMC10514463; doi:10.1016/j.isci.2023.107809)
Supplement: Document S1. Figures S1–S3 and Tables S1–S5 [file mmc1.pdf]

iScience, Volume *26*

Supplemental information

Gut flora alterations among aquatic firefly *Aquatica leii* inhabiting various dissolved oxygen in fresh water

Zi-Shun Zhao, Lin-Yu Yang, Fu-Xin Li, Wei Cun, Xing-Yan Wang, Cheng-Quan Cao, and Qi-Lin Zhang

Table S1. Raw and clean data of the V4-V5 fragment of 16S rRNA gene generated in this study. Related to Figure 4.

Sample name	Raw data (Mb)	Tag number	Base number	Mean length	OTU number	Chao1	ACE	Shanno	Simpson	Coverage (%)
Hyperoxia_1	22.4	215541	87792105	407.31	90	290.62	273.82	1.58	0.42	0.99
Hyperoxia_2	22.4	167421	68893672	411.50	99	303.84	295.65	1.91	0.36	0.99
Hyperoxia_3	25.9	190583	77397227	406.11	85	345.23	367.96	1.72	0.44	0.99
Hyperoxia_4	29.2	218267	89113068	408.28	78	308.01	301.48	1.63	0.46	0.99
Hyperoxia_5	26.4	195178	79766076	408.68	75	534.78	533.25	2.52	0.39	0.99
Hyperoxia_6	21.6	149788	61952010	413.60	65	311.47	326.57	1.51	0.41	0.99
Hyperoxia_7	23.4	175151	72356095	413.11	63	295.93	276.38	2.31	0.31	0.99
Hyperoxia_8	24.6	187211	76870507	410.61	60	322.36	354.79	2.29	0.33	0.99
Hyperoxia_9	24.9	198580	80845105	407.12	59	260.14	265.31	2.15	0.37	0.99
Hyperoxia_10	21.2	193411	78898627	407.93	80	274.42	284.32	1.67	0.45	0.99
Hypoxia_1	23.3	184392	76322505	413.91	58	250.38	236.03	1.96	0.29	0.99
Hypoxia_2	26.2	196000	80604624	411.25	52	260.95	278.34	1.91	0.33	0.99
Hypoxia_3	23.7	176904	73870534	417.57	50	281.68	295.85	2.23	0.20	0.99
Hypoxia_4	24.4	179846	75369304	419.08	47	270.43	288.72	2.35	0.17	0.99
Hypoxia_5	23.0	178244	74158924	416.05	45	223.15	227.42	2.01	0.22	0.99
Hypoxia_6	27.4	162910	66327595	407.14	44	210.48	195.36	1.28	0.65	0.99
Hypoxia_7	19.3	165308	67670871	409.36	42	258.61	280.62	1.43	0.51	0.99
Hypoxia_8	23.0	192155	79077115	411.53	41	279.05	296.38	1.88	0.39	0.99
Hypoxia_9	30.1	219920	91325732	415.27	41	352.23	345.73	2.47	0.18	0.99
Hypoxia_10	24.7	179762	74331264	413.50	54	201.74	191.59	2.64	0.21	0.99
Normoxia_1	26.4	179438	75489789	420.70	40	424.46	426.73	3.15	0.11	0.99
Normoxia_2	29.1	153306	64375993	419.92	37	283.43	291.46	3.03	0.13	0.99
Normoxia_3	19.6	177983	72648196	408.17	37	234.91	237.17	1.94	0.39	0.99
Normoxia_4	21.1	139520	58430417	418.80	36	139.87	157.97	2.37	0.21	0.99

Normoxia_5	24.0	183255	75633356	412.72	35	153.36	165.25	2.08	0.33	0.99
Normoxia_6	22.1	173108	70904834	409.60	34	131.53	148.13	1.70	0.46	0.99
Normoxia_7	19.3	163136	66393791	406.98	33	189.83	160.22	1.76	0.45	0.99
Normoxia_8	25.2	189742	78605091	414.27	33	130.28	139.45	2.41	0.18	0.99
Normoxia_9	23.7	172462	71172395	412.68	32	366.53	354.68	1.91	0.35	0.99
Normoxia_10	19.6	173365	70141799	404.59	39	159.72	172.63	1.57	0.49	0.99

Table S2. Summary of gut microbiota at various taxonomy based on 16S rRNA gene amplicon. Related to Figure 2 and S2.

Sample	Phylum	Class	Order	Family	Genus	Species
Hypoxia_1	5	7	15	18	19	10
Hypoxia_2	5	7	15	18	18	10
Hypoxia_3	5	7	15	20	20	10
Hypoxia_4	5	7	15	21	21	10
Hypoxia_5	5	7	15	20	20	11
Hypoxia_6	5	7	13	21	22	9
Hypoxia_7	5	7	15	20	19	10
Hypoxia_8	5	7	15	19	21	9
Hypoxia_9	5	7	15	19	20	10
Hypoxia_10	5	7	14	18	20	9
Normoxia_1	5	7	16	20	20	12
Normoxia_2	5	7	16	21	19	10
Normoxia_3	5	7	15	22	20	10
Normoxia_4	5	6	13	19	19	11
Normoxia_5	5	7	15	18	18	10
Normoxia_6	5	7	16	20	20	9
Normoxia_7	5	7	15	22	21	10
Normoxia_8	5	7	15	19	20	10
Normoxia_9	5	7	16	18	19	9
Normoxia_10	5	7	15	22	21	10
Hyperoxia_1	5	7	14	21	20	9
Hyperoxia_2	5	7	15	18	19	9
Hyperoxia_3	5	7	16	20	18	10

Hyperoxia_4	5	7	16	19	19	10
Hyperoxia_5	5	7	16	18	18	9
Hyperoxia_6	5	7	15	18	18	9
Hyperoxia_7	5	7	16	19	21	10
Hyperoxia_8	5	7	16	19	19	9
Hyperoxia_9	5	7	16	19	20	10
Hyperoxia_10	5	7	15	22	20	9

Table S3. The linear discriminant analysis of the effect size was verified by *FDR*, and the *P* value was verified. Related to Figure 3.

Species	Group	Mean	*FDR*
Order__Enterobacterales	Normoxia	4.51	0.004
Family__Enterobacteriaceae	Normoxia	4.45	0.009
Genus__*Cronobacter*	Normoxia	4.48	0.016
Genus__*Aquicella*	Normoxia	4.12	0.013
Class__Gammaproteobacteria	Hypoxia	5.39	0.012
Family__Pseudomonadaceae	Hypoxia	4.99	0.004
Genus__*Pseudomonas*	Hypoxia	4.96	0.006
Order__Pseudomonadales	Hypoxia	4.77	0.005
Family__Alcaligenaceae	Hypoxia	4.39	0.033
Genus__*Pseudoxanthomonas*	Hypoxia	4.37	0.023
Genus__*Mycobacterium*	Hyperoxia	4.45	0.001
Order__Corynebacteriales	Hyperoxia	5.25	0.002
Family__Mycobacteriaceae	Hyperoxia	4.45	0.001
Family__Nocardiaceae	Hyperoxia	4.78	0.004
Genus__*Rhodococcus*	Hyperoxia	4.73	0.002

Table S4. Summary of sequencing data from metagenomics. Related to STAR Methods.

Sample name	Hypoxia_ 1	Hypoxia_ 2	Hypoxia_ 3	Normoxia_ 1	Normoxia_ 2	Normoxia_ 3	Hyperoxia_ 1	Hyperoxia_ 2	Hyperoxia_ 3	Average
Clean base (bp)	14.78	13.16	12.77	12.54	12.76	13.24	12.52	12.61	12.75	13.02
Contig number	1384992	1223895	1304173	1263287	1322900	1342390	1284413	1330813	1333083	1309994
N50 (bp)	752	844	743	716	710	725	659	703	750	733.56
N90 (bp)	357	366	359	356	356	356	352	355	360	357.44
Max (bp)	40426	75831	40425	40425	40427	40425	221399	40426	40425	64467.67
Min (bp)	300	300	300	300	300	300	300	300	300	300.00
Average Size (bp)	20363	38065.5	20362.5	20362.5	20363.5	20362.5	110849.5	20363	20362.5	32383.833

Table S5. Differential function of gut flora among three DOC groups based on KEGG annotation. Related to Figure 8.

Level 1	Level 3	*FDR* values
Metabolism	Drug metabolism - other enzymes	0.0291
	Pentose and glucuronate interconversions	0.0488
	Drug metabolism - cytochrome P450	0.0445
	Ascorbate and aldarate metabolism	0.0133
	Retinol metabolism	0.0209
	Steroid hormone biosynthesis	0.0388
	Steroid biosynthesis	0.0321
	Glycosphingolipid biosynthesis - globo and isoglobo series	0.0157
	Chlorocyclohexane and chlorobenzene degradation	0.0285
Organismal Systems	Pancreatic secretion	0.0399
	Bile secretion	0.0404
	Hematopoietic cell lineage	0.0344
Genetic Information Processing	DNA replication	0.0434
Environmental Information Processing	Neuroactive ligand-receptor interaction	0.0027

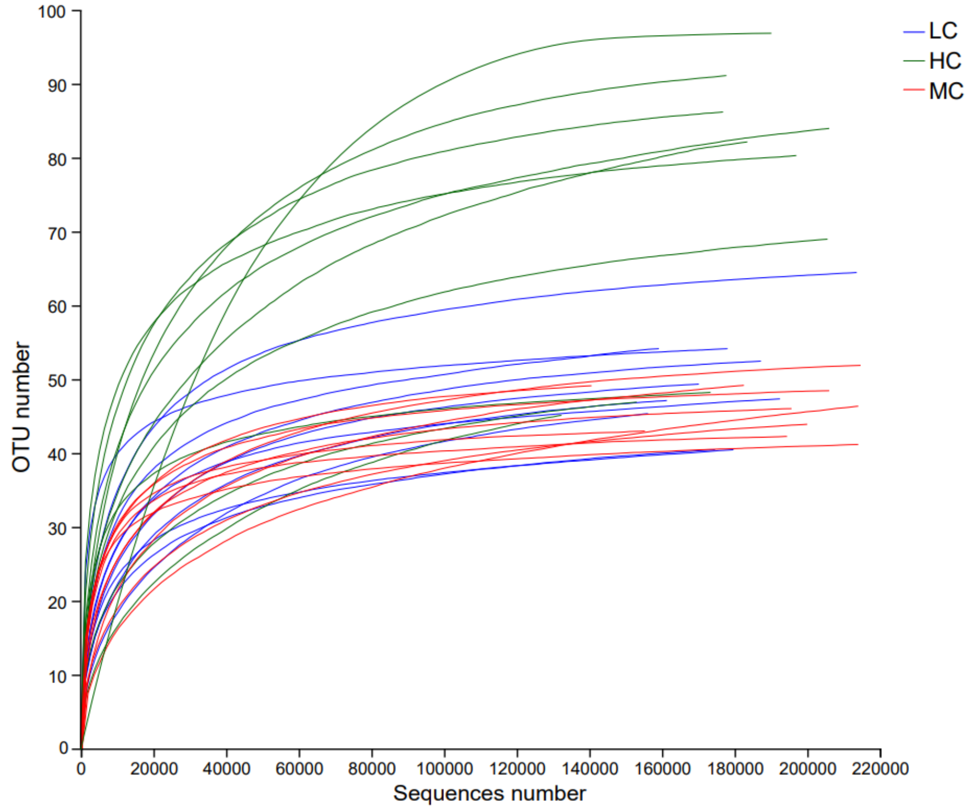

Figure S1. Rarefaction curves of gut samples of *A. leii* sequencing of bacterial communities. Color-coded lines represent different samples: Blue line represents samples in the hypoxic group; red line represents samples in the normoxic group; green line represents samples in the hypoxic group. Related to STAR Methods.

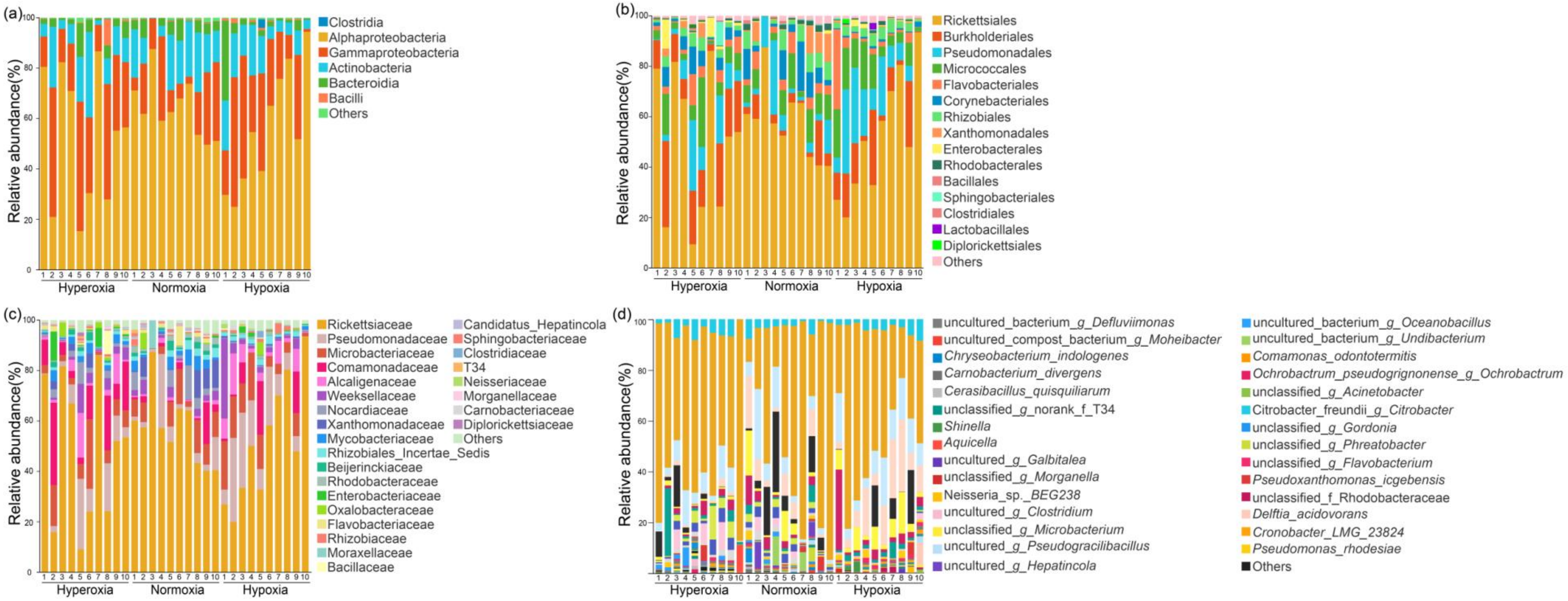

Figure S2. The relative abundance of bacterial taxa at the level of (a) class, (b) order, (c) family and (d) species for three DOC groups based on 16S rRNA gene amplicons. Related to Figure 2.

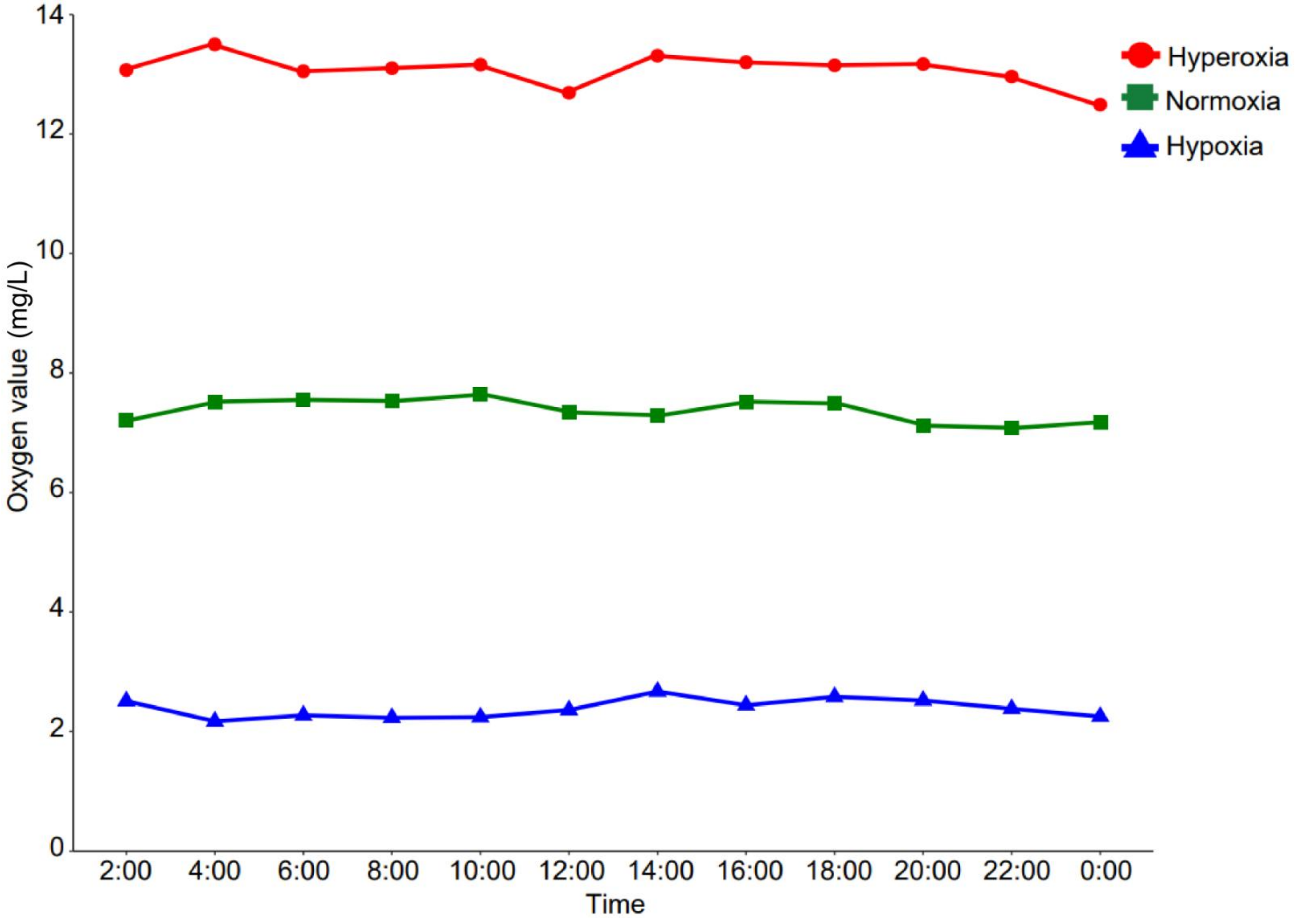
Hyperoxia
Normoxia
Hypoxia
Oxygen value (mg/L)
Time
0
2
4
6
8
10
12
14
2:00
4:00
6:00
8:00
10:00
12:00
14:00
16:00
18:00
20:00
22:00
0:00

Figure S3. Oxygen distribution in 24 hours, the different colors of the lines represent the different groups, the horizontal coordinates indicate the oxygen concentration monitored every two hours, and the vertical coordinates indicate the amount of oxygen in the water (mg/mL). hypoxia: 2.50 $\pm$0.50 mg/L, normoxia: 7.00 $\pm$0.50 mg/L, hyperoxia: 13.00 $\pm$0.50 mg/L. Related to STAR Methods.
